# Metagenomic and Phenotypic Insights Into Biofilm‐Forming Pathogens in Patients With Nosocomial Sepsis

**DOI:** 10.1155/bmri/8989667

**Published:** 2026-04-20

**Authors:** Haleema Sadia, Arshia Amin, Iftikhar Ahmed

**Affiliations:** ^1^ Department of Bioinformatics and Biosciences, Capital University of Science and Technology, Islamabad, Pakistan, cust.edu.pk; ^2^ National Culture Collection of Pakistan (NCCP), Land Resources Research Institute (LRRI), National Agricultural Research Centre, Islamabad, Pakistan, parc.gov.pk

**Keywords:** 16S rDNA sequencing, biofilm-related infections, metagenomic analysis, microbial diversity, polymicrobial pathogens, virulence factor

## Abstract

Biofilm‐related infections significantly contribute to bacterial diseases, with estimates suggesting that at least 80% of such infections are associated with biofilms. These infections often involve opportunistic pathogens, which not only influence the type of infection but also impact the microenvironment by interacting with other polymicrobial pathogens, thereby altering microbial diversity within the infection site. The present study was designed to assess potential changes in bacterial communities across various infection types. The 50 samples were collected and pooled from different anatomical locations: II‐H1 (calf), ul‐H2 (thighs), ft‐H3 (upper leg), ct‐H4 (chest), and Ca‐H5 (catheter). The 16S rDNA sequencing was performed on 10 representative samples using the Sanger method to identify bacterial taxa, whereas the metagenomic analysis was conducted on the Illumina MiSeq platform (Illumina, Inc., San Diego, California). Sanger sequencing identifying several bacterial strains including *Bacterium* MS‐AsIII‐61, *Bacterium HB33*‐1, *Mammaliicoccus sciuri* SSB38, multiple *Staphylococcus* species (*S. aureus* DA101 and S8, *Staphylococcus* sp. C0021‐01R and TSA25S, *S. cohnii* FC2265, and S. *saprophyticus* A), and *Enterobacter hormaechei* D15. The metagenomics analysis revealed variations and diversity in the different location across the organ by relative abundance of 5 bacterial phyla and 38 species. The *Proteobacteria* phylum was the most abundant phylum across all sites, with the highest prevalence observed in Ca‐H5, followed by ul‐H2, ct‐H4, II‐H1, and ft‐H3 in the decreasing order. In contrast, the *Bacteroidetes* phylum exhibited the highest abundance in ft‐H3. Catheter‐associated infections (Ca‐H5 site) show a homogeneous ARG profile, dominated by genes supporting biofilm formation and persistence. MSA samples reflect diversity in methicillin and multidrug resistance genes, consistent with surgical‐site and opportunistic infections. Trypto samples may represent an environmental or experimental condition leading to alternative ARG expression, highlighting site‐ or condition‐specific variations. The different virulence factor responsible for the boost in the establishment of biofilms in these pathogens includes, surface adhesion proteins, increasing resilience to environmental, efflux pumps, quorum‐sensing regulators, stresses, and antibiotic treatments. The study demonstrates the dynamic nature and impact of biofilm‐related infections at anatomical sites. It also focused on biofilm‐associated infections at surgical sites, their progression into chronic conditions, and the corresponding treatment patterns. The integration of metagenomic analysis with phenotypic studies provided deeper insights into the roles of key genes and their mechanisms in biofilm formation.

**Highlights of the Study**

➢ The role of nonculturable bacteria, which are mostly neglected in routine hospital tests, showed greater antibiotic resistance.
➢ The importance of key processes such as quorum sensing and adhesion and their contribution to biofilm formation and resilience.
➢ Some most specific gene and their potential for biofilm formation to develop the therapeutic targets.
➢ These findings highlighted the limitations of current diagnostic tools in detecting resistant bacteria within biofilms.
➢ To better understand biofilm formation and the effectiveness of phenotypic and metagenomics approaches.

## 1. Introduction

The rate of hospital‐acquired or nosocomial infections prevalence rose by 25% and up to 15% in underdeveloped and developed countries, respectively. This rise in prevalence resulted in mortality of around 40,000 hospitalized patients round the globe [1]. The reason behind the increase in incidence of nosocomial infections among patients who are under treatment in hospitals is the insertion of medical devices which include a variety of central venous catheters (CVCs) utilized in their therapy and different procedures like minor surgeries, tumor treatments, and hemodialysis [2]. Catheterization procedures which cause the nosocomial infections are mostly related to antimicrobial‐resistant microorganisms, including numerous *Staphylococcus* spp., enterobacterial species, *Enterococcus* spp., and fungi such as *Candida* spp. [3]. The colonization of the matrix is by multiple species of bacteria. These multiple species synchronize and communicate with each other and construct a microenvironment that is different from that of planktonic bacteria. The different bacterial populations have different microenvironments which have different properties; some are aerobic and others are mixed, which make the bacteria resistant up to 1000 times more than planktonic to common drugs. This is partially elucidated by the slow cell development and reduced metabolic activity of these biofilms [4]. Moreover, the EPS matrix surrounding biofilms, which can account for 50%–90% of their total biomass, inhibits antimicrobial agents from penetrating biofilms [5]. During infection, the bacteria are considered important constituents of the microbial community.

The bacterial type and concentration can be responsible for further information about the infection and contamination source. Moreover, determining the role of the bacterial community in infection could help in determining the effect and role of opportunistic pathogens in infection. In order to better understand the microbial load in the community assembly and functionality, metagenomics studies can be a better approach to provide better insight of existed microbes. Several studies have utilized metagenomic sequencing to explore the microbial composition of biofilms in nosocomial settings. For instance, Alotaibi et al. [6] employed 16S rRNA sequencing to analyze bacterial communities in hospital wastewater, identifying opportunistic pathogens commonly linked to healthcare‐associated infections. Similarly, Banar et al. [7] reported the prevalence and resistance genes of *Stenotrophomonas maltophilia* from clinical biofilm‐forming isolates using metagenomics, highlighting challenges in treatment. Other investigations have focused on catheter‐associated infections, revealing complex microbial consortia dominated by *Proteobacteria* and *Firmicutes*, with extensive multidrug resistance [2, 8]. These studies underscore the power of metagenomics to uncover nonculturable, drug‐resistant species involved in biofilm formation, which traditional culturing methods often miss. Metagenomics is considered a source to determine the microbiota′s native constitution by utilizing the DNA directly extracted from a control sample with normal microbiota so as to evaluate the native microbial structure [9]. To increase the availability of metagenomics investigations through targeted metagenomics, next‐generation sequencing (NGS) has been used [10].

Over the last decade, there have been substantial advances in NGS technologies that have made it possible to use metagenomic sequencing for infection diagnosis. Significant reductions in cost and turnaround time coupled with increased data yields have enabled research and clinical laboratories to begin using metagenomic‐based approaches for infection diagnosis, surveillance and outbreak management. Clinical metagenomics (CMg), which refers to the use of metagenomics to sequence all microbial and host genetic material present in patient samples for the diagnosis of infectious diseases [11, 12]. According to the WHO (2016), the prevalence of diabetes is increasing rapidly in low‐ and middle‐income countries, where complications such as nonhealing ulcers place a significant economic and social burden on patients. In these regions, reliance on conventional culturing methods often provides a limited view of microbial diversity and dominance patterns [13].

Miller et al. [14] studied that the metagenomics allow us for profiling of whole community structure and identifying individual microbes without the need for isolation, offering a scalable and high‐throughput method to monitor hospital environments.

Hendriksen and Munk et al. [15] applied large‐scale shotgun metagenomics to untreated sewage samples across multiple countries, including LMICs. Their work demonstrated resistome profiling as a feasible tool for global surveillance, linking ARG prevalence with antibiotic usage and socioeconomic factors.

In the present study, variable regions of 16Sr RNA gene and metagenomic analysis determine the sources of secondary infections among different patients admitted to a tertiary care public sector hospital. Five different organs, including the upper leg, lower leg, foot, chest, and chest, were selected to investigate the potential variation in bacterial communities and biofilm infection in relation to different organs. The present findings could help in better understanding biofilm infection as well as bacterial aerobic and anaerobic diversity, which changes from person to person and from location to location.

Studying polymicrobial biofilms is crucial, particularly in the context of nosocomial infections, as they present significant challenges in resource‐limited healthcare settings. These biofilms complicate treatment due to their enhanced resistance to antibiotics and their ability to persist in hostile environments. In many low‐resource hospitals, routine diagnostics rely on culture‐based methods, which often fail to detect slow‐growing, anaerobic, or nonculturable organisms, as well as their resistance genes [10]. This diagnostic limitation delays effective therapy and increases the risk of treatment failure. Metagenomic approaches overcome these constraints by enabling culture‐independent detection of microbial communities and associated resistance mechanisms directly from clinical samples, offering rapid and comprehensive diagnostic insights. This is especially valuable in under‐resourced settings, where conventional diagnostics are either unavailable or insufficient to manage complex biofilm‐associated infections [11]. Also, NGS has been successful in resource‐limited situations where the integration of metagenomic approaches with phenotypic studies provides a deeper understanding of the genetic forces responsible for biofilm formation. It ultimately provides more successful and accurate intervention in challenging clinical settings. Lowering sequencing costs and securing sustainable funding, including utilizing investments made during the pandemic, are crucial. Global organizations and trade agreements play a vital role in minimizing supply chain challenges. Equally important is developing local metagenomics capacity to promote decentralized surveillance systems, which enable early detection and response to infectious diseases at their origin, thereby strengthening global health security.

The current study hypothesized that metagenomic analysis could be helpful in the detection of resistance and virulence genes that contribute to the chronic and treatment‐challenging nature of the biofilm‐associated infections. The aim of this study was to investigate the microbial composition, antibiotic resistance, and biofilm‐forming potential of pathogens involved in nosocomial sepsis. The main objective of the current research work was to evaluate the biofilm‐forming ability of clinical isolates using both qualitative and quantitative methods—specifically, the test tube method for preliminary detection of biofilm formation and the microtiter plate assay for accurate quantification of biofilm biomass. Furthermore, the key biofilm‐related genes and their gene expression were analyzed in order to provide a comprehensive understanding of the microbial and molecular mechanisms underlying surgical site biofilm–associated infections.

## 2. Materials and Methods

### 2.1. Sample Collection

**Figure 1:**
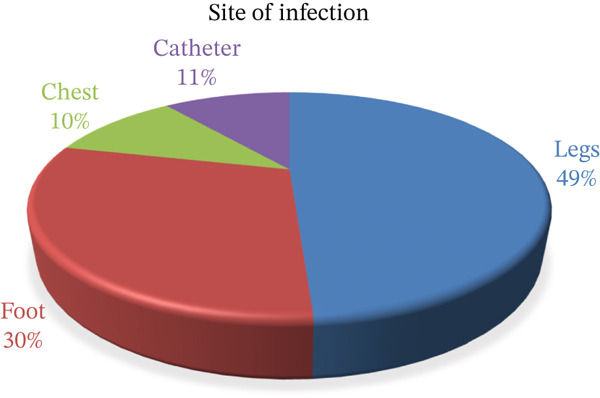
The distribution of sampling across the different body parts.

A total of 50 swab samples were taken from patients in the ward of the orthopedic and general surgical ward in a tertiary care hospital located in Pakistan during November and December 2022 (Figure 1). To reduce the cost of metagenomic sequencing, a total of 50 tissue samples were pooled from debridement tissue. From every site, two samples were collected: One was used for metagenomic analysis, and the duplicate was used for isolation in the laboratory as well as for Sanger sequencing. According to hospital records, most cases are reported during this season, indicating a temporal peak in infections. All the samples are collected from debridement tissue by using sterile techniques, and the wound must be thoroughly cleansed prior to sampling to avoid contamination and obtain accurate microbiological results. A short sampling window represents a limitation of this study, as potential seasonal variations in microbial composition and resistance profiles could not be assessed. Moreover, ward‐level factors and within‐hospital variability were not controlled, which may influence the generalizability of the findings. The swabs contained transportation material for all types of microorganisms. All swabs were stored in an icebox and transported to the university laboratory. The swabs taken from the patients were categorized based on the organ of infection, as they were collected from the foot, upper leg (thigh), lower leg, and chest to determine the infection and diversity of bacteria. Notably, the samples of infection collected were labeled p1–p50.

Anatomical sites were chosen based on their relevance to the study objectives, prevalence of biofilm‐associated infections, and accessibility for sampling. The common site may include indwelling catheters, the area disposed to the biofilm formation because of the compromised tissue integrity, surgical wounds, and prolonged exposure to medical devices. The strict aseptic technique was used during the sampling and transport to avoid contamination. This includes the use of sterile equipment and containers as well as the disinfection of the skin surface by using appropriate antiseptics on the sampling site. To ensure the proper technique and minimize handling errors, the samples were collected by trained personnel. To preserve microbial viability and avoid contamination from the environment, the samples were kept in sterile conditions, mostly temperature‐controlled, during transport. Amies or Stuart′s transport media (semisolid media) were used to maintain the viability of aerobic bacteria during transit to the laboratory.

Moreover, to ensure the traceability and integrity throughout the study, the clear labeling and documentation of each sample is ensured. These steps are important during the sampling to ensure the reliability of result and reducing the risk of introducing extraneous microorganisms. The proper ethical approval was obtained by university as well as the administrative authority of hospital (Bioethical Committee of Faculty of Health and Life Sciences,CUST, Sp23‐BSBE‐04). The hospital ethics committee approved the study. All participants provided informed consent prior to their involvement in the study, ensuring compliance with ethical standards and respect for individual autonomy. At the site‐level, some metagenomic samples were obtained as pooled data (e.g., multiple wound swabs combined to represent a single infection site) for community‐level analysis, whereas the isolate‐level assays (biofilm formation and resistance profiling) were conducted on 20 clinical isolates. Each biofilm assay was performed in triplicate (*n* = 3 biological replicates per isolate), and resistance profiling in duplicate, to ensure reproducibility.

### 2.2. Culture‐Dependent Method Isolation and Morphological Identification

In the culture‐dependent method the swab was plated on the mannitol salt agar (HiMedia Laboratories, India), chocolate agar (Oxoid Ltd., United Kingdom), blood agar (HiMedia Laboratories, India), and tryptone soya agar (Oxoid Ltd., United Kingdom). The plates were kept in the incubation at 37°C for 24 h in aerobic conditions. The single colony was obtained by streaking and pure colonies were used for further analysis. In order to determine the morphology of bacteria the precise observation was made for determining the margin, elevation, texture, size, and light transmission. The diversity of strains was obtained by different morphologies and the same morphology was considered to be a single strain [16].

DNA was extracted from bacterial cultures by suspending colonies grown on agar media in 1% Phosphate buffered saline (PBS), followed by vortex mixing for homogenization. A 200‐*μ*L aliquot was centrifuged at 13,000 rpm for 5 min to form a pellet, which was then lysed with 500 *μ*L of a buffer containing 1‐M sucrose, 1.5‐M Tris‐HCl, 1‐M EDTA, 2% SDS, 0.05‐M MgCl2, and 10‐*μ*L proteinase K, with incubation at 56°C for 2 h. Following lysis, phenol (250 *μ*L) and chloroform: isoamyl alcohol (250 *μ*L) were added, and the mixture centrifuged at 13,000 rpm for 10 min. The aqueous phase was recovered for DNA precipitation using chilled isopropanol and sodium acetate, incubated at −20°C for 2 h, centrifuged at high speed for 10 min, washed with 70% ethanol, air‐dried, and resuspended in 60‐*μ*L TE buffer. DNA quality and quantity were confirmed by Nanodrop with 260/280 ratios between 1.7 and 1.9 and concentrations of 800–1200 ng/*μ*L. Integrity was verified by electrophoresis on a 1% agarose gel containing ethidium bromide, run at 75 V and 500 mA for 60 min and visualized under a UV transilluminator using BioDoc Analyzer. PCR amplification targeted the near full‐length 16S rRNA gene (~1300 bp) using 0.6 *μ*L each of forward and reverse primers (10 *μ*M, final 0.2 *μ*M), 0.6‐*μ*L dNTPs (10 mM, final 0.2 mM), 3 *μ*L PCR buffer (10X), 3 *μ*L MgCl2 (25 mM, final 2.5 mM), 0.6‐*μ*L Taq polymerase (5 U/*μ*L, final 3 U), 3‐*μ*L template DNA, and nuclease‐free water to 30‐*μ*L total volume, performed on a Galaxy XP Thermal Cycler (BIOER) under optimized cycling conditions. PCR products were purified by magnetic bead cleanup, followed by a two‐step library preparation for Illumina MiSeq sequencing involving initial amplification with locus‐specific primers containing Illumina overhang adapters, and subsequent enrichment with limited‐cycle PCR to attach multiplexing indices and sequencing adapters. Final libraries were quantified, pooled, and sequenced using MiSeq v3 reagents with a PhiX spike‐in control. This method provides high‐quality sequencing of near full‐length 16S rRNA amplicons suitable for microbial identification and community profiling.

### 2.3. Screening of Biofilm‐Forming Bacteria

The capacity of pure strains to form biofilm formation was checked by the microliter plate reader and test tube method. A single bacterial colony of each isolate was inoculated into 3‐mL tryptone soy broth (TSB) and incubated at 37°C for 24 h. After 24 h, 2 mL of 2% glucose was added in each TSB test tube and was again incubated for 24 h at same temperature After total incubation of 48 h, TSB growth media was discarded, and the test tubes were washed thrice aseptically with PBS of pH 7.4 to remove any unbound bacteria. After washing with PBS, the remaining attached microbes were fixed by using 3 mL of 99% methanol and tubes were left for 15 min. In order to observe biofilm production by microorganisms, each test tube was stained with 0.3 mL of 0.1% crystal violet (CV) for 5–6 min. After 5–6 min, the test tubes were washed carefully with running tap water in order to remove the excess stain. Once the excess stain was removed, the tubes were left to dry by placing them in inverted position. After drying, the adherent cells were solubilized with 1.5 mL of 33% (*v*/*v*) glacial acetic acid, and optical density (OD) was measured at 620 nm using a spectrophotometer (Spectro quant Prove 300 plus [Model 1]). The blank for each test tube was PBS. The scores of biofilm production were determined as follows: nonbiofilm former (OD_620_ < 0.275), weak biofilm former (0.275 ≤ OD_620_ < 0.55), medium biofilm former (0.55 ≤ OD_620_ < 0.825), and strong biofilm former (0.825 ≤ OD_620_). Each test was carried out in duplicate, and the average OD was taken. The quantitative estimation of the biofilm formation was performed by the microtiter plate and spectrophotometer assay as described by Furtuna et al. [17].

The isolates were grown in TSB and incubated overnight at 37°C in a static condition to obtain sufficient microbial growth. After 24 h incubation, the cultures were standardized with the same medium and 150 *μ*L of each of the cultures were inoculated in the 96‐well plates (Linbro) in triplicates. The 96‐well plates were then incubated at 37°C for 48 h in a static condition. After incubation, the wells were drained and gently washed in three trays of PBS (PBS pH 7.3) to remove free‐floating planktonic cells. The plates were then stained with 0.1% (*w*/*v*) CV solution and allowed to stand for 15 min. The plates were allowed to dry in an inverted form. The wells of the microtiter plates were destained with 150 *μ*L of 95% ethanol–acetic acid and allowed to stand for 15 min. Enzyme‐linked immunosorbent assay (ELISA) autoplate reader (Labsystems Type 355) was used to measure the OD at wavelength of 620 nm. These OD values were considered as an index of attachment to surface. The experiment was performed in triplicates and the mean values were considered as an index of biofilm formation.

### 2.4. Gene Expression Analysis by RT‐PCR

The study included isolates from various bacterial strains, but primarily focused on *Staphylococcus* species including *Staphylococcus aureus* strains DA101 and S8, as well as other *Staphylococcus* species such as *Staphylococcus* sp. strain C0021‐01R, TSA25S, *Staphylococcus cohnii*, and *Staphylococcus saprophyticus*. Other bacterial isolates like *Bacterium MS-AsIII-61*, *Bacterium strain HB33-1*, *Mammaliicoccus sciuri*, and *Enterobacter hormaechei* were likely included as outgroup controls for comparison. The primers used in the study were genus‐specific, designed to amplify sequences unique to *Staphylococcus*, allowing precise identification and differentiation among the *Staphylococcus* isolates. These were not common universal primers but targeted specific conserved regions within the genus, facilitating focused study on *Staphylococcus* species while excluding unrelated bacteria.

Polymerase chain reactions were performed on a Galaxy XP Thermal Cycler (BIOER, PRC). 2X Syber Green (intercalating dye) (Bilrt Germany) was used to perform RT‐PCR.

RT‐PCR was accomplished to strengthen the specific DNA sequences by primer sets for gyrase B, clumping factor A (*clfA*), *and* fibronectin‐binding protein A (*FNBA*). The annealing temperature of each primer set was optimized through gradient PCR. The reaction mixtures for each PCR mixture contained 1 *μ*L of cDNA template, 0.2 *μ*L each of forward and reverse primers (10‐*μ*M stock, 0.2‐*μ*M working concentration), 0.2 *μ*L of dNTPs (10‐mM stock, 0.2‐mM working concentration), 1 *μ*L of 10X PCR buffer, 1 *μ*L of MgCl2 (25‐mM stock, 2.5‐mM working concentration), 0.3 *μ*L of Taq polymerase (5‐U/*μ*L, 1.5‐U final), and 6.1‐*μ*L of PCR water for a total volume of 10 *μ*L. The optimized PCR conditions were as follows: initial denaturation at 95°C for 10 min; 40 cycles of denaturation at 95°C for 1 min, annealing at optimized temperatures for 1 min, and extension at 72°C for 1 min; a final extension at 72°C for 10 min; and a hold at 4°C. Three primer sets were used to optimize the annealing temperature (Ta). Sequences of forward and reverse primers are as follows.

For gene *gyrB* primers (Tm 62°C, product size 300 bp) included CGATTGGGCGTGGCTTTCAGC‐forward and CGATTGGGCGTGGCTTTCAGC‐reverse sequences, *clfA* gene (Tm 65.9°C and product size 72 bp) included GTAGACCCCCCGGCAGAGT‐forward and CGATTGGGCGTGGCTTTCAGC‐reverse sequences, *FNBA* gene (Tm 58.5°C and product size 130 bp) included ATCAGCAGATGTAGCGGAAG‐forward and ATCAGCAGATGTAGCGGAAG‐reverse sequences.

The *gyrase B* primers used were optimized at 58.8°C, *clfA* at 61.9°C, and *FNBA* at 60°C. PCR products were confirmed via gel electrophoresis. For RT‐PCR, the reaction mixture included 1 *μ*L of cDNA template, 0.2 *μ*L each of forward and reverse primers (10‐*μ*M stock, 0.2‐*μ*M working concentration), 5 *μ*L of 2X Syber Green, and 3.6 *μ*L of nuclease‐free water for a total volume of 10 *μ*L, and the reaction was performed on a Mic PCR system with similar thermal cycling conditions [18].

The housekeeping genes functioned as internal controls to normalize the expression levels of target genes across different samples. Furthermore, standard curves were generated using known concentrations of cDNA to evaluate the efficiency and specificity of the qPCR assays. These measures enhance the reliability and comparability of the results, supporting accurate interpretations of gene expression levels in the context of polymicrobial biofilm infections. A scanning electron diagram of the biofilm was generated with a JSM IT200. JICA, Version 2. With an image size of 1280 × 960, color Mode 1 and a field of view of 38.79–29.09 *μ*m were used. The field size ranged from 128–96.

### 2.5. Statistical Analysis

Statistical analysis was performed using SPSS software, employing *t*‐tests to compare group means. A *p* value threshold of 0.05 was used to determine statistical significance, with *p* < 0.05 indicating a significant difference between different strains. The Bayesian re‐estimation approach was applied to obtain accurate species abundance estimates from metagenomic sequencing data, improving resolution by probabilistically redistributing ambiguously classified reads, whereas the Pearson’s chi‐square test on resistance profile data (*χ* ^2^ = 46.8, df = 12, *p* = 5.76) indicated statistically significant differences in resistance patterns among strains.

### 2.6. Isolation via a Culture‐Independent Method

#### 2.6.1. DNA Extraction From Bacterial Wound Culture Samples

DNA extraction was carried out by the CTAB method. This method is useful for isolating high‐quality DNA, which is useful for further molecular processing [19]. The quality of the DNA was assessed by the NanoDrop ratio. The DNA band was visualized in a 1% agarose gel and compared with a 1‐kb ladder. Total DNA was used for the construction of DNA libraries. For sequencing, the extracted DNA was subjected to high‐throughput sequencing on an Illumina platform (MiSeq), using paired‐end sequencing with read lengths typically ranging from 150 to 300 base pairs. Paired‐end sequencing was employed to improve alignment accuracy and facilitate detection of structural variants and repetitive regions. The combination of the modified CTAB extraction and Illumina paired‐end sequencing enabled the generation of high‐quality genomic data suitable for downstream applications such as genome assembly, gene annotation, and phylogenetic analyses. The high‐quality DNA sequence libraries used in this study have been deposited in the NCBI Sequence Read Archive (SRA), and the corresponding sample‐level accession numbers are provided in Table S2.

#### 2.6.2. Quality Control

FastQC was used to check the quality of the raw sequencing reads, whereas Fastp was used to trim and filter the reads. Host reads were removed by alignment to the GRCh38 assembly to eliminate human contamination, ensuring cleaner data for analysis. MEGAHIT is employed for metagenome assembly, providing essential contig statistics such as N50 and L50, and Bracken estimates species abundance based on classifications from Kraken2.

Kraken 2 was used to assign taxonomic information to the reads in the curated database [8]. Kraken 2 is the newest version of Kraken and is a taxonomic classification system in which exact k‐mer matches are used to achieve high accuracy and fast classification speeds. A prebuilt database in Kraken 2, named the MiniKraken database (containing bacterial, viral, and archaeal sequences), was used for the alignment of reads and assignment of taxonomic information to the reads.

#### 2.6.3. Abundance Estimation

The Bayesian re‐estimation is a statistical method which estimates the abundance of species in DNA from metagenomic samples by computing methods. In the Kraken, the abundance of species does not estimate but classifies reads to the best matching location in the taxonomic tree. In order to estimate the abundance at the species level, the genus or above, we use the Kraken database itself to know the probabilities of sequences that indicate how much of the sequence from each genome is identical to the other genome in the database. The Kraken classifier and Bracken both combined to produce accurate species‐ and genus‐level abundance estimates even when a sample contains two or more near‐identical species [6, 20].

### 2.7. Generation of a Complete Taxonomic Hierarchy

#### 2.7.1. Kraken‐Biom

The program takes as input one or more files output from the Kraken report tool [20]. Each file was parsed, and the counts for each OTU (operational taxonomic unit) were recorded, along with the database ID (e.g., NCBI) and lineage. The extracted data are subsequently stored in a BIOM table, where each count is linked to the sample and the OTU to which it belongs. This BIOM format is generated mainly for importing abundances and taxonomic hierarchies to R for further exploration of microbiome data.

### 2.8. Diversity Calculation and Visualization

#### 2.8.1. *α* Diversity

Diversity can be represented only as richness (i.e., the number of different species in an environment); otherwise, diversity can be measured considering the abundance of the species in the environment (i.e., the number of individuals of each species inside the environment). To measure *α* diversity, different indices, such as the Shannon, Simpson, and Chao1 indices, can be used.

#### 2.8.2. Beta Diversity

Beta diversity analysis was performed to evaluate the compositional dissimilarities between the five microbial community samples (G1–G5). The feature abundance table was processed in R using the phyloseq and vegan packages. Two distance metrics, that is, Jaccard index (presence/absence‐based) and Bray–Curtis dissimilarity (abundance‐based) were applied. Principal coordinates analysis (PCoA) was conducted on each distance matrix using the cmdscale function. The first two principal coordinates (PC1 and PC2) were extracted to visualize sample clustering patterns in two‐dimensional space.

#### 2.8.3. Comprehensive Analysis of Microbial Strains From Metagenomic Data

Using high‐throughput sequencing and computational approaches, sequences are matched to databases of reference genomes to identify bacterial strains at species or strain levels. Techniques such as k‐mer based genome‐specific markers (GSMs) or assembly‐free approaches allow rapid and accurate strain detection, even at low abundance, by targeting unique regions of each genome.

#### 2.8.4. Computational Approach for Virulence Gene Identification

Virulence factors were identified from the metagenomic sequencing data by mapping high‐quality reads against a curated virulence factor gene database using dedicated bioinformatics tools with strict identity and coverage thresholds to ensure accurate detection.

## 3. Results

### 3.1. Morphological Identification of Isolates

The study included both male and female patients. A greater number of male patients exceeded female patients were included in the study overall, as male patient admissions to the wards were greater than female patient. Affected areas for sampling included the calf, thighs, foot, chest, and catheter installations across different patient IDs (Table 1). Each entry included the unique identifier (ID) and the corresponding site of surgery or infection, highlighting areas, that is, ll for calf, ul for upper leg (thigh), ft for foot, ct for chest and ca for catheter.

**Table 1:** Sample ID and site of postsurgical infection.

Sr. No.	ID	Site of surgery/infection
1	ll‐H1	Calf
2	ul‐H2	Thighs
3	ft‐H3	Foot
4	ct‐H4	Chest
5	ca‐H5	Catheter′s installed

**Figure 2:**
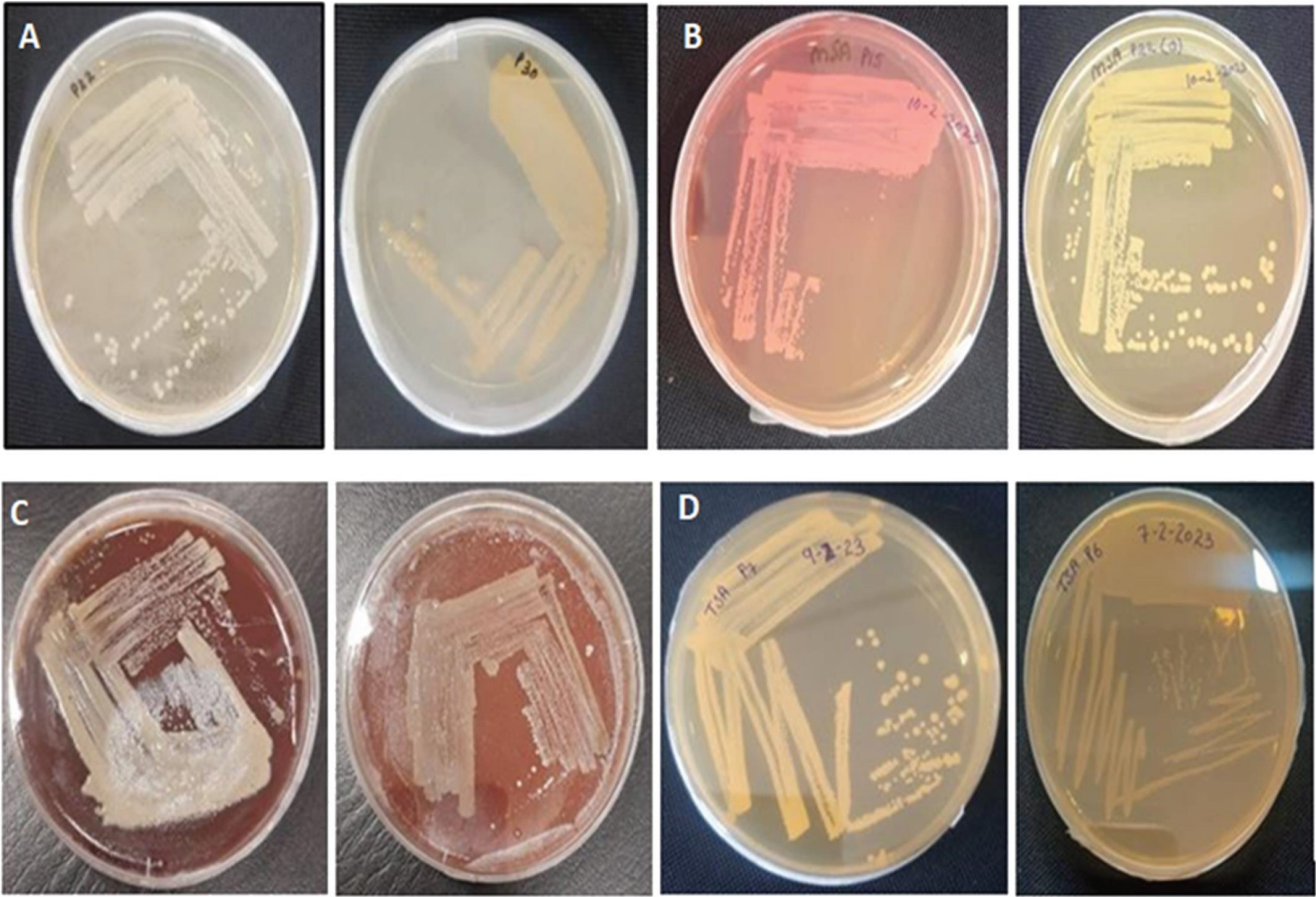
Colonies from distinct samples or experimental conditions. The variations in colony morphology and agar color reflect the use of different media types for bacterial growth and isolation: (A) nutrient agar, (B) mannitol salt agar, (C) blood agar, and (D) tryptone soya agar.

This information was crucial for understanding the distribution of surgical interventions and infection locations within the study population. Furthermore, additional insights have been mentioned Figure 2 and as Table 2 which included details isolated colonies, morphological characteristics of various pathogens cultured on different media, pathogen IDs, and specific colony features. These characteristics were crucial for the identification and differentiation of bacterial species for valuable insights into their potential pathogenicity and behavior in clinical environments. The 14 strong biofilm‐forming strains were selected after measurement of OD at 620 nm (Figure 3). Both test tube (Figure 3a) and the microtiter plate methods (Figure 3b) revealed significant differences in biofilm biomass with *p* < 0.05.

**Table 2:** This table provides a detailed description of the various pathogens, including the media used for their growth and specific colony characteristics in laboratory.

S. No.	Strain ID		Media	Colony shape	Margin (edge)	Elevation	Color	Texture	Light transmission
1	P5	*Enterobacter hormaechei* strain D15	TSA	Round	Undulate	Raised	Milky white	Shiny	Translucent
2	P6	*Mammaliicoccus sciuri* strain SSB38	TSA	Circular	Entire	Flat	Golden	Shiny	Opaque
3	P7	*Staphylococcus aureus* strain DA101	TSA	Round	Entire	Flat	White	Smooth	Translucent
4	P22	*Staphylococcus* sp. strain C0021‐01R	MSA	Circular	Rhizoid	Raised	Milky white	Shiny	Translucent
5	P26(a)	*Staphylococcus* sp. TSA25S	MSA	Circular	Entire	Raised	Yellow	Shiny	Translucent
6	P27	*S*. *aureus* strain S8	MSA	Irregular	Lobate	Flat	White	Shiny	Opaque
7	P29	*Staphylococcus cohnii* strain FC2265	MSA	Circular	Entire	Raised	Yellow	Shiny	Translucent
8	P18	*Staphylococcus saprophyticus* strain_n_A	NA	Circular	Lobate	Flat	Yellow	Smooth	Opaque
9	P5	Bacterium strain HB33‐1	NA	Irregular	Lobate	Raise	White	Shiny	Opaque
10	P30	Bacterium MS‐AsIII‐61	NA	Circular	Entire	Flat	White	Smooth	Opaque

### 3.2. Antimicrobial Resistance

The interplay between biofilm formation and antimicrobial resistance is critical, as biofilms not only enhance bacterial survival by limiting antibiotic penetration and promoting genetic adaptations but also contribute to the emergence of multidrug‐resistant strains, complicating infection management and treatment efforts.

**Figure 4:**
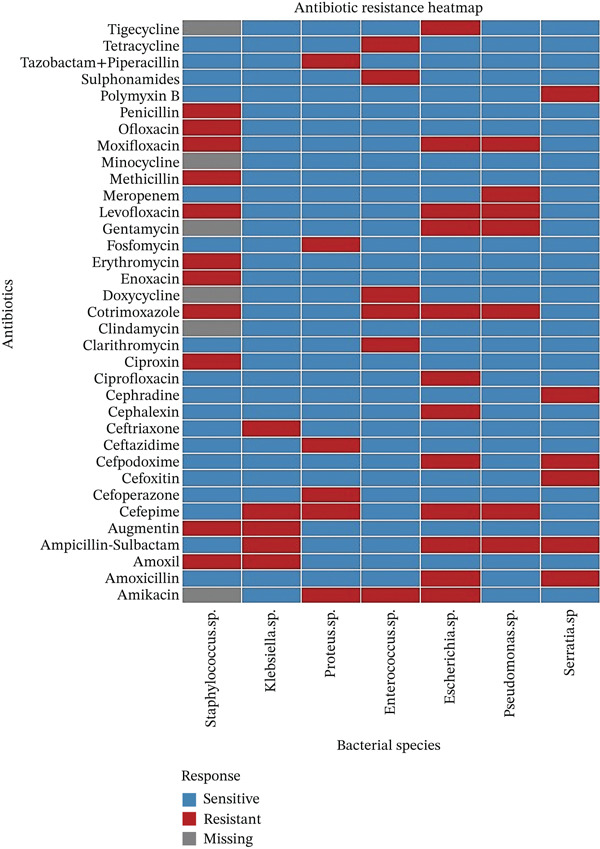
Heatmap showing antibiotic resistance (red), sensitivity (blue), and missing data (gray) across bacterial species tested against multiple antibiotics.

The data of resistance (indicated by “R”) of *S*. *aureus*, *Klebsiella* spp., *Proteus spp.*, *Enterobacter spp.*, *Escherichia coli*, *Pseudomonas spp.*, and *Serratia* spp. to various antibiotics (Table S1) revealed widespread multidrug resistance across these species, especially to beta‐lactams, fluoroquinolones, and aminoglycosides, underscoring the challenges in treating infections caused by these pathogens. The data indicated widespread multidrug resistance among these bacterial species, which limits the effectiveness of common antibiotics and highlights the need for careful antibiotic selection and susceptibility testing. The high resistance to beta‐lactams, fluoroquinolones, and aminoglycosides across multiple species underscores the challenge of treating infections caused by these organisms, especially in cases of hospital‐acquired infections or infections in immuno‐compromised patients (Figure 4).

### 3.3. Gene Expression Analysis

**Figure 5:**
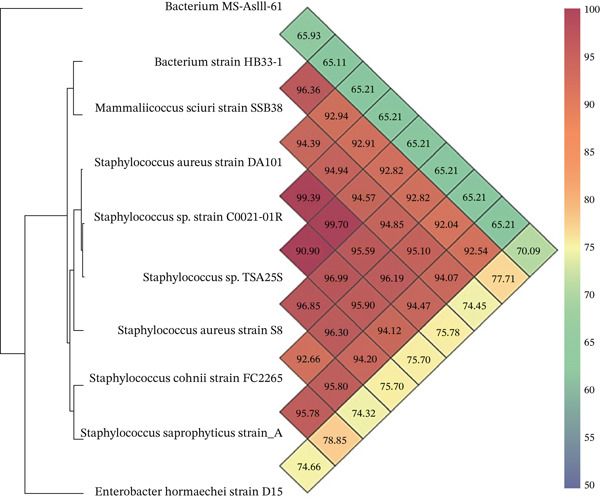
Heatmap and dendrogram showing pairwise average nucleotide identity (ANI) values among bacterial strains. Higher ANI values (red) indicate close genomic relatedness, whereas lower values (green/blue) reflect distant relationships; the clustering visually groups similar strains.

The genetic similarities among various bacterial strains, particularly within the *Staphylococcus* genus, were shown as color gradients in Figure 5, with green (lower similarity) to red (higher similarity). *Staphylococcus* species such as *S*. *aureus* and *Staphylococcus* sp. showed high genetic similarity and clustered closely together with percentages in the mid to high 90 s, suggesting that they were closely related. In contrast, *the MS-AsIII*‐61 bacterium, the HB33‐1 bacterial strain, and the D15 *E*. *hormaechei* strain exhibited lower similarity (approximately 65%–75%) with the *Staphylococcus* strains. These results indicated that these genes were more genetically distant. This heatmap effectively highlighted both the close genetic relationships within the *Staphylococcu*s group and the more distant relationships with other bacterial strains.

**Figure 6:**
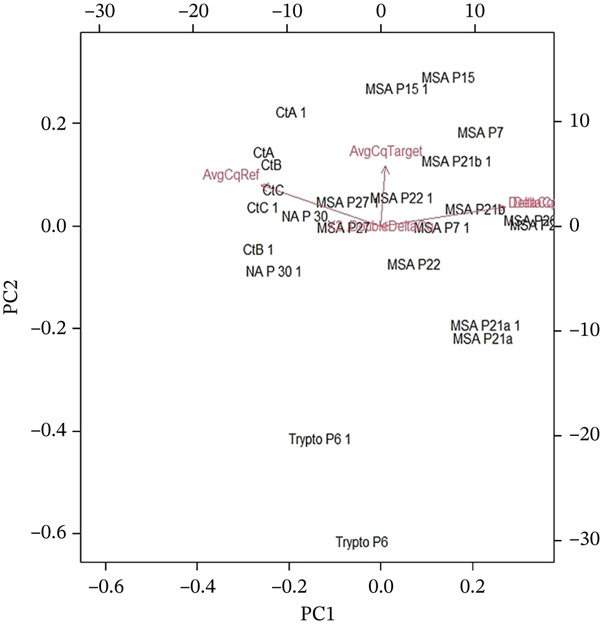
Principal component analysis (PCA) biplot showing the distribution of samples based on PC1 and PC2, with vectors indicating the influence of qPCR variables (e.g. AvgCqRef, and AvgCq Target). The plot shows clustering of bacterial communities based on antibiotic resistance gene (ARG) profiles across different sampling sites.

The clustering of bacterial communities based on antibiotic resistance gene (ARG) profiles across different sampling sites was estimated by PCA analysis. The *x*‐axis (PC1) and *y*‐axis (PC2) represent the first two principal components (Figure 6). Each labeled point shows a sample from a specific anatomical site (e.g., Ct = catheter, MSA = methicillin − susceptible *S*.*a* *u* *r* *e* *u* *s* group). Red vectors (arrows) show variables related to gene expression for resistance markers such as AvgCqTarget, AvgCqRef, and DeltaCq. The above mentioned vectors indicate the orientation and strength of association between samples and ARG profiles.

The observed clusterings indicated that the catheter (CtA, CtB, and CtC) samples clustered close near the center, which indicated that they shared a similar ARG profile. MSA‐related samples (MSA P15, MSA P7, MSA P21, MSA P22, etc.) grouped more toward the right side (positive PC1 values), which showed a distinct ARG pattern compared with catheter samples. NA P30 grouped with the catheter samples, which suggested overlapping gene resistance characteristics. Trypto samples (P6 and P6‐1) were separated toward the bottom negative PC2 values, which indicated that they harbored different resistance gene compositions, distinct from both catheter and MSA clusters. The separation along PC1 suggested that methicillin‐related resistance markers (in the MSA group) were the primary drivers of variability. The catheter samples likely reflected ARGs related to biofilm‐associated resistance (efflux pumps and adhesion proteins) which clustered them tightly. The Trypto group′s separation indicated the unique ARGs which probably linked to metabolic adaptation or stress response genes. Their connection with the classical methicillin resistance determinants was not very apparent. The arrows (AvgCqTarget, DeltaCq) pointed toward the MSA cluster, suggesting that quantitative gene expression values of ARGs (like mecA, efflux pump regulators, or quorum sensing genes) drove the variance in these samples.

In order to understand the role of fibronectin‐binding protein A (*FNBA*) gene, their molecular mechanism in the biofilm formation and antibiotics resistance, the rtPCR was performed, which showed the role in adhesions which were important for the bacterial adherence and colonization. In Figure 7 it was showed that the gene expression levels of the *FNBA* (red bar) and gyrase genes (black bar) were high. These observations suggested that the *FNBA* gene tended to be more actively expressed in the majority of samples than the Gyrase gene. *FNBA* is expressed at higher levels than the gyrase gene because it could be a key adhesin protein involved in *S*. *aureus* infection. *FNBA* could mediate bacterial adhesion to host extracellular matrix proteins like fibronectin, facilitating colonization, internalization into host cells, and biofilm formation. That protein played a crucial role in host–pathogen interaction and immune evasion. Its elevated expression supported the bacteria′s ability to infect and persist in host tissues, unlike the gyrase gene, which was a housekeeping gene generally expressed at stable levels to maintain basic cellular functions.

**Figure 8:**
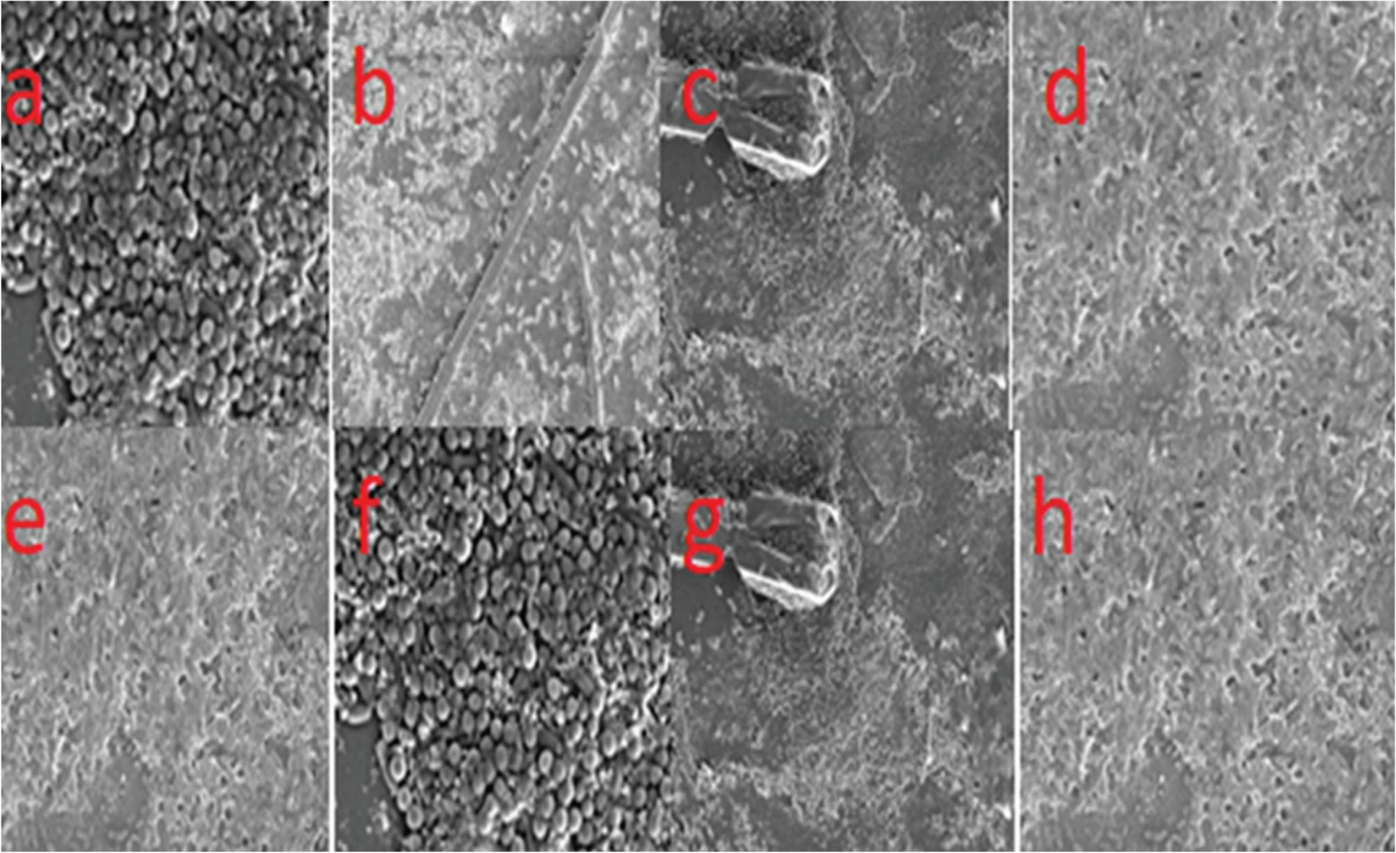
Images (a) and (f) show dense clusters of round or cocci‐shaped bacterial cells, indicative of biofilm formation. In contrast, Images (b) and (e) reveal a sparser distribution of cells, possibly representing a different growth phase or environmental condition. Images (c) and (g) highlight a fibrous structure interacting with the bacterial cells, suggesting adherence to a foreign object or material. Finally, Images (d) and (h) depict surfaces with minimal bacterial presence, potentially due to treatment or conditions that reduce cell attachment.

The gene expression levels of two genes, gyrase and *clfA*, across various samples and the controls showed that the overall, *clfA* expression (red bars) was consistently greater than gyrase gene expression (black bars) across all samples (Figure 7b). *clfA* is highly expressed compared with the gyrase gene because it could play a crucial role as a virulence factor in *S*. *aureus. clfA* as was reported to be a cell surface protein that promoted the bacterial adhesion to host proteins like fibrinogen, facilitating colonization, biofilm formation, and immune evasion [21, 22]. Evidences of varying intensities of biofilms were also seen through microscopy (Figure 8). Its expression was often upregulated to enhance bacterial attachment and survival, especially under conditions of mechanical stress or infection. In contrast, *gyrA*, the gyrase gene, was reported to be a housekeeping gene that maintained essential cellular functions but could not vary extensively in expression. Therefore, *clfA* expression could be greater as it was actively involved in pathogenic processes, whereas *gyrA* serves as a stable control gene [23].

### 3.4. Metagenomic Analysis

Details of wound infection samples collected from postsurgical patients in orthopedic and surgery wards with corresponding metadata and accession numbers are provided in Table S2. The broader microbiome analysis (Figure 9a) illustrated the dominance of *Proteobacteria* in ca‐H5, followed by ul‐H2, ct‐H4, ll‐H1, and ft‐H3 in descending order. In addition, the abundance of *Bacteroidetes* in ft‐H3 surpassed that in ct‐H4 and ca‐H5 but was not detected in ll‐H1 or ul‐H2. The third most abundant phylum, *Firmicutes,* exhibited relatively higher levels in ll‐H1 and ul‐H2; however, it was present in smaller quantities in ft‐H3 and ct‐H4. Notably, *Actinobacteria* exhibited lower abundances in ll‐H1, ul‐H2, and ct‐H4 than in the other strains, but no differences were observed in ft‐H3 or ca‐H5. The presence of the phylum Chordata in ll‐H1 and ft‐H3 was likely due to contamination. The diagram showed the five most abundant phyla across samples from ll‐H1 to ca‐H5 collected from distinct human organ locations.

A total of 38 species were identified across all the samples in Figure 9b. Regarding species abundance, *Pseudomonas aeruginosa* was abundant in all the samples, with a relatively greater presence in the ct‐H4 samples than in the other samples. *Argentinense* sp. were present in II‐H1, ul‐H2, and ct‐H4, gradually increasing in ca‐H5, and was notably absent in ft‐H3. *E. coli* was found in II‐H1, ul‐H2, ft‐H3, and ct‐H4, whereas it was not detected in ca‐H5. *Flavum* sp. were exclusively present in ft‐H3 and were absent in all the other samples. *Insolitus* sp. were observed in ul‐H2, ct‐H4, and ca‐H5. *Maribilis* sp. were present in ul‐H5 and ca‐H5. *Mendocina sp.* were identified in ll‐H1, ul‐H2, and ft‐H3, with a slight increase in ca‐H5 and absence in ct‐H4. *Sapiens* sp. exhibited an abundance in ll‐H1, ft‐H3, and ct‐H4; were less prevalent in Ca‐H5; and were not found in ul‐H2. *Ovatus* sp. were abundant in ul‐H2, and *G. monteilii* were exclusively present in ft‐H3. *Trematum* sp. were noted in ll‐H1, ft‐H3, and Ca‐H5. *S*. *aureus* was abundant in ll‐H1, ul‐H2, ct‐H4, and Ca‐H5. *Diphtheriae* sp. were identified in ll‐H1, ul‐H2, and ct‐H4. *Xylanisolvens* sp. were abundant in ll‐H1, ul‐H2, and ft‐H3 but were not present in ct‐H4 or Ca‐H5. *monteilii* sp. were observed in ft‐H3.

The distinct spatial distributions of the test samples by the Jaccard and Bray–Curtis metrics showed that the G3 (ftH3) and G4 (ctH4) had marked divergence from the remaining samples and proved to be major representatives of the community‐level variation (Figure 10a whereas G1 (llH1) and G2 (ulH2) clustered closely, indicating community level similarities (Figure 10).

The most prominent species in the in ll‐H1 and ul‐H2, which occupied a greater portion, was *P. aeruginosa* (teal color). In the other samples, species belonging to *E*. *coli* (brown) and *S*. *aureus* (pink) also appeared but with varying abundances. In samples ft‐H3, ct‐H4, and ca‐H5, species diversity was greater, where a mixture including *Morganella morganii* (gray) and *Streptococcus pneumoniae* was more evenly distributed. The diversity observed in samples ft‐H3, ct‐H4, and ca‐H5 contained a balanced mix of species, which could be further understood by examining their respective characteristics, such as GC content and aerotolerance (Table 3). The data revealed diverse genomic GC contents and metabolic adaptabilities, including facultative anaerobes and aerobic organisms, obligate aerobes, highlighting their potential roles in biofilm formation and infection persistence. In biofilms, the availability of aerotolerant and anaerobic bacteria was reported to be an indicator of established biofilms, and the aerotolerant anaerobes and anaerobes could exist in biofilms and that the spatial distribution of oxygen gradients could influence biofilm structure and composition, and it was an indicator of how biofilms under low‐oxygen or anaerobic microenvironments tend to harbor more tolerant or resistant populations [24].

**Table 3:** A list of aerobic, facultative, and obligate anaerobic strains identified from test samples.

Genus species	Percentage of GC	Aerotolerance
*Achromobacter ruhlandii*	20%	Aerobic
*Achromobacter*_*xylosoxidans*	15%	Aerobic
*Acinetobacter*_*baumannii*_TYTH‐	18%	Aerobic
*Stenotrophomonas maltophilia*	2%	Aerobic
*Serratia*_*marcescens*	29%	Facultative anaerobic
*Staphylococcus*_*aureus*_subsp._*aureus*_Tager_104 2820837	12%	Unknown
*A*. *baumannii* TYTH‐1	21%	Obligate aerobe
*Achromobacter insolitus*	20%	Aerobe
*Proteus mirabilis*	20%	Facultative anaerobe
*Pseudomonas aeruginosa*	32%	Facultative aerobe
*S*. *maltophilia*	6%	Aerobic
*Escherichia coli*	9%	Facultative anaerobic
*Klebsiella pneumoniae*	35%	Facultative anaerobic
*Morganella morganii* subsp. *morganii* KT	15%	Facultative anaerobic
*S*. *marcescens*	12%	Facultative anaerobic
*A*. *baumannii* TYTH‐1	8%	Obligate aerobe
*Massilia oculi*	1%	Aerobic
*Bordetella trematum*	20	Aerobic
*Myroides* sp. ZB35	17	Obligated aerobic

*Achromobacter ruhlandii*, *Serratia* sp. *SSNIH1, Achromobacter xylosoxidans*, *Acinetobacter_baumannii_TYTH, S*. *maltophilia*, *Serratia_marcescens*, and *Staphylococcus_aureus*_subsp._aureus_Tager_104 2820837 were among the abundant bacterial species. The role of specific bacterial genes in biofilm formation—a key mechanism enhancing bacterial persistence and resistance. The involvement of various bacterial genes in biofilm formation highlighted a key factor in bacterial persistence and resistance (Table 4).

**Table 4:** Important virulence factors and their role in biofilm formation.

Strain	Gene	Role in biofilm formation
*Acinetobacter baumannii*	adeF	Membrane‐fusion protein (AdeFGH efflux pump [VF0504]–biofilm [VFC0271])
	bfmR	Biofilm‐controlling response regulator (BfmRS [VF0463] –regulation [VFC0301])
	csuB	Csu pilus subunit CsuB (Csu fimbriae [VF0461]–biofilm [VFC0271])
	adeH	Outer membrane protein (AdeFGH efflux pump [VF0504]–Biofilm [VFC0271])
	abaR	DNA‐binding HTH domain‐containing protein (quorom sensing [VF0471]–biofilm [VFC0271])
	adeG	Cation/multidrug efflux pump (AdeFGH efflux pump [VF0504]–biofilm [VFC0271])
	bfmR	Biofilm‐controlling response regulator (BfmRS [VF0463]–regulation [VFC0301])
	bap	Biofilm‐associated protein (bap [VF0462]–biofilm [VFC0271])

*Pseudomonas aeruginosa*	alg44	Alginate biosynthesis protein Alg8 (alginate [VF0091]–biofilm [VFC0271])
	mucE	Small envelope protein MucE (alginate [VF0091]–biofilm [VFC0271])
	hdtS	1‐acyl‐sn‐glycerol‐3‐phosphate acyltransferase (Acylhomoserine lactone synthase [VF0907]–biofilm [VFC0271])
	mucA	Alkaline metalloproteinase precursor (alginate [VF0091]–biofilm [VFC0271])
	lasI	Autoinducer synthesis protein lasI (quorum sensing [VF0093]–biofilm [VFC0271])
	cupB3	Usher CupB3 (CupB fimbriae [VF0925]–biofilm [VFC0271])
	rhlR	Transcriptional regulator RhlR (quorum sensing [VF0093]–biofilm [VFC0271])

*Staphylococcus aureus*	hlgB	Gamma‐hemolysin component B (‐hemolysin [VF0011]–exotoxin [VFC0235])
	SAV_RS00935	O‐antigen ligase family protein (capsule [VF0003]–immune modulation [VFC0258])
	icaR	Ica operon transcriptional regulator IcaR (intercellular adhesion proteins [VF0014]–biofilm [VFC0271])
	icaB	N‐deacetylase, involved in polysaccharide intercellular adhesin (PIA) synthesis (intercellular adhesion proteins [VF0014]–biofilm [VFC0271])
	hlb	Beta‐hemolysin (beta‐hemolysin [VF0002]–exotoxin [VFC0235])
	clfA	Clumping factor A, fibrinogen‐binding protein (clumping factor [VF0004]–adherence [VFC0001])
	sasC	LPXTG‐anchored repetitive surface protein SasC (SasC [VF1010]–biofilm [VFC0271])
	aae	Autolysin/adhesin Aae (SE2319 [VF1000]–adherence [VFC0001])
	rhlR	Transcriptional regulator RhlR (quorum sensing [VF0093]–biofilm [VFC0271])

*Klebsiella pneumoniae*	mrkB	Fimbrial chaperone protein mrkB precursor (Type 3 fimbriae [VF0567]–biofilm [VFC0271])

*Escherichia coli*	cahS	Ag43/Cah family autotransporter adhesin (Cah, AIDA‐I type [VF1129]–Biofilm [VFC0271])

*P*. *aeruginosa* was also among abundant species in all test samples, likely due to its well‐known role as a versatile, opportunistic pathogen capable of thriving in diverse environments and forming resilient biofilms. It was reported to be a major contributor to persistent infections and resistances. *S*. *aureus* was notably abundant in samples ll‐H1, ul‐H2, ct‐H4, and ca‐H5, which reflected its common involvement in a wide range of infections and its ability to colonize various anatomical sites. The dominance of these species could be attributed to their virulence factors, adaptability, and capacity to evade host defenses, which could make them prevalent in clinical infections.

Few of the bacterial species which were observed in the sepsis samples and biofilms include *A. baumannii*, which was observed to have relied on genes such as *adeF*, *adeG*, and *adeH*, which were part of the AdeFGH efflux pump and contributed to both antibiotic resistance and biofilm formation. Other genes, such as *bfmR* and *bap*, regulated biofilm formation and provided structural integrity. *P*. *aeruginosa* reported to utilize genes such as *alg44* and *mucE* for alginate production, which form a protective biofilm matrix, whereas *lasI* and *rhlR* were involved in quorum sensing and coordinating biofilm development.

*S*. *aureus*, which was observed in high OTUs, utilizes genes *icaR* and *icaB* to synthesize polysaccharides, which are essential for biofilm structure, along with other adhesion genes, such as *clfA* and *sasC*. *K. pneumoniae* and *E. coli* are also reported to utilize fimbrial proteins such as *mrkB* and autotransporter adhesins such as *cahS* to anchor themselves to surfaces, facilitating biofilm formation. Collectively, these genes could enhance the ability of bacteria to form biofilms, increasing their resistance to environmental stresses and antibiotic treatment [25].

*A*. *xylosoxidans*, which was an opportunistic pathogen that had been isolated from hospital‐associated infections, such as surgical site infections (SSIs). Initially, it had low virulence ability with sporadic elimination; however, later, it was identified to have more virulence factors with severe infection and, in some cases, fetal outcomes [26]. These bacteria were also involved in coping with biofilm‐type infections to prevent antibiotic treatment. This pathogen could transfer resistance genes and increase tolerance to antimicrobial agents. Biofilm‐type infections developed in patients with deep infections caused by prostheses or implants [27]. Therefore, the prevention and treatment of SSIs were challenging and could be prevented by the intrinsic nature of these bacteria.

#### 3.4.1. *Acinetobacter johnsonii*

*Acinetobacter* spp., aerobic and nonfermentative which were mostly present in the diverse environment including human skin, soil, water and sludge [28]. These bacteria had been responsible for severe hospital‐acquired infections, which highlights their clinical importance [29]. *A. johnsonii* could adjust its phenotype when it was grown as a biofilm and could change its metabolism under antibiotic stress, providing implications for subsequent biofilm control [30].

#### 3.4.2. *Bordetella trematum*

*B*. *trematum*, a Gram‐negative, oxidase‐negative, motile nonspore‐forming, capsulated rod which grow at a temperature range of 25°C–42°C [31, 32]. Associated infection could occur in wounds and could be treated by removal via debridement to reduce the chance of reoccurrence into nonviable tissue. However, the pathogenicity of these microorganisms has been unclear, as they could be new emerging microorganisms [33]; therefore, investigations of their pathogenicity, virulence factors, and important genes were needed to improve treatment and antimicrobial therapy. The *B. trematum* was most commonly isolated from the necrotic lesions or site with impaired tissue perfusion such as chronic ulcer however many reports showed its cause bacteremia and even sepsis by invading into the bloodstreams [34].

Among three clinical isolates of *B. trematum*, none could cause invasive disease. The antimicrobial therapy was used for the treatment of *B. trematum*, but the choice of surgical debridement was also a good option in chronic ulcer. Generally, polymicrobial infections of chronic wounds were best addressed by an interdisciplinary approach involving surgeons as well as microbiologists.

Chronic, nonhealing wounds were frequently characterized by hypoxic cell death, tissue necrosis, and an impaired immune response, all of which were linked to decreased tissue perfusion; these conditions created ideal growth conditions for a large number of microorganisms, thus explaining the polymicrobial nature of such infections [35]. The microorganisms from the many sources could adhere to and colonize the unusual habitat and made an interaction with that environment and played a significant role in the wound pathogenesis and healing [35, 36].

#### 3.4.3. *Bacteroides fragilis*

*B*. *fragilis has been* the human colon microorganism but could also cause colitis and other inflammatory bowel diseases (IBDs) of anaerobic opportunists. The enterotoxigenic *B*. *fragilis* (ETBF) strain of *B. fragilis* produces the toxin fragilysin (Bft), which is associated with acute diarrheal disease, IBD, and colorectal cancer (CRC) [37].

#### 3.4.4. *A. baumanii* TYTH‐1

*A*. *baumannii*, an obligate aerobe coccobacillus, was one of the most prevalent causes of nosocomial infections [38]. MDR *A*. *baumannii* was an emerging opportunistic pathogen when in biofilms and was associated with a variety of nosocomial pulmonary infections, bacteremia, SSIs, secondary meningitis, and urinary tract infections.

#### 3.4.5. *S*. *maltophilia*

*S*. *maltophilia*, the only known species of *Stenotrophomonas* that caused opportunistic infections in humans. *S. maltophilia* could cause infections of the soft tissue, pneumonia, sepsis, endocarditis, osteochondritis, mastoiditis, and meningitis [7].

#### 3.4.6. *Kerstersia gyiorum*

*K*. *gyiorum* has been isolated from many human sepsis samples. The word “gyiorum,” which was also derived “from the limbs,” was given when the type strain was identified and characterized by Coenye et al. [39] from lower‐extremity wounds.

Overall polymicrobial infections were becoming increasingly prevalent in clinical cases and were becoming a global health concern. Limited knowledge has been available about the factors inducing their pathogenesis. Several factors which had been identified for the formation of biofilms and enhanced pathogenesis included: presence of outer membrane protein A (OmpA), phospholipids, EPS, capsules, a siderophore‐mediated iron acquisition system, and phospholipases [40].

Key virulence factors from important bacterial strains such as *A*. *baumannii*, *P*. *aeruginosa*, *S*. *aureus*, *Klebsiella pneumoniae*, and *E*. *coli*, emphasizing their crucial roles in biofilm formation has been highlighted in Table 4. These factors included membrane proteins (e.g., adeF and adeH) and adhesins (e.g., bap and icaB) which contribute to biofilm matrix production, bacterial surface attachment, contribute to multidrug resistance, but also enhances biofilm development, linking resistance with persistence [41]. Response regulators like bfmR could control biofilm development, whereas quorum sensing components (abaR, lasI, and rhlR) coordinate community behavior within biofilms. Pili and fimbriae subunits (csuB, cupB3, and mrkB) had been responsible for denhanced microbial adherence and biofilm structural integrity [42, 43].

In pathogens like *S*. *aureus*, exotoxins (hlgB and hlb) and surface proteins (*clfA* and sasC) facilitate biofilm resilience and immune evasion [43]. The presence of these virulence genes collectively could support bacterial colonization, persistence, and resistance within biofilm communities, highlighting their significance in infection processes and potential targets for therapeutic intervention (Table 5).

These virulence determinants were identified from metagenomic datasets using multiple bioinformatics pipelines and databases (e.g., VFDB, CARD, ResFinder), which cross‐validated the results and increased the reliability of the findings. The consistent detection of those genes across different annotation platforms strengthened the evidence for their role in biofilm development and persistence in clinical infections.

## 4. Discussion

Out of the 50 samples, 7 were female and 43 were male; the males were infected by different infections and developed biofilms after surgery. The swab samples were collected from different wards, such as orthopedic and general surgical ward patients (49%, leg; 30%, foot; 10%, chest; and 10%, chest). The initiation date of infection and the date of hospital admission were recorded as acute or chronic infection. The presence of necrotic tissue, inflammation, and red granules and the long duration of the wound can only affect whether the patient has biofilms because there is no direct method for detecting chronic infection. A history of different diseases is a major factor for biofilm infection.

From the 50 samples, up to 30 strains on different media were revived aerobically. Some samples cannot be removed because of a lack of anaerobic chambers or because of secondary infection caused by fungal infection. The strains that were revived on the different media were identified by morphology. The incidence of biofilm infection due to accidents is greater in males than in females because the exposure of males to the environment is greater than that of females. The infection in a patient is chronic as a result of a long‐duration road accident, catheter implant, postsurgical infection, abrasion, or chronic injury from nosocomial opportunity pathogens. Our findings are similar to those of Fernandes and Dias [44], who reported that males were more likely to have PAIs (76%) because of few challenging jobs which make them more prone to accidents. These fractures expose soft tissue to hematoma formation and ultimately wound contamination.

The quantitative estimation of biofilms was performed using a test tube nephrometer. The OD was recorded at 620 nm. Among the 14 samples, p2, p6, p7, p9, p10, p13, and p18 were nonbiofilm producers; p4, p5, p8, and p30 were medium biofilm producers; and p42 and p45 were strong biofilm producers. This can also be confirmed by visualizing the layer on the test tube. An examination of the biofilms that were detached from the samples revealed that the biofilms harbored groups of microbial communities. The comparison of biofilm formation in test tubes with that in microtiter plates was also performed with the same procedure. Of the 14 strains, 6 showed weak, 5 moderate, and 2 strong biofilm formation. Weak biofilm formation was interpreted as 0–< 0.2, moderate biofilm formation was interpreted as 0.2–0.4, and strong biofilm formation was interpreted as > 0.4. In this study, moderate results were categorized as positive (Figure 8).

The PCA of ARG profiles revealed clear partitioning of samples by anatomical site: catheter‐associated samples clustered tightly and were distinct from the MSA (*Staphylococcus*‐dominated) group and the Trypto samples, whereas the ft‐H3 (upper leg) samples showed a relative shift toward *Bacteroidetes*‐associated resistome signatures. These results reflected that catheter‐associated biofilms were commonly polymicrobial and enriched for *Proteobacteria* and mobile ARGs, whereas wound‐ and skin‐associated communities contained higher proportions of *Bacteroidetes* and SSIs were frequently dominated by *Staphylococcus* spp. with methicillin and multidrug‐resistance determinants [45–47].

The tight clustering of catheter samples and their alignment with vectors for quantitative gene measures (AvgCqTarget / DeltaCq) was consistent with the role of biofilm‐associated features—including EPS formation, persister cell development, and horizontal gene transfer—in accumulating ARGs and were responsible for producing reproducible resistance signatures across catheter samples. Recent multiomics and metaproteomic studies emphasize that biofilms amplify phenotypic resistance and facilitate ARG exchange [48, 49].

The separation of MSA samples along PC1 reflected the dominance of *Staphylococcus* species and associated ARGs (such as *mecA* and efflux/aminoglycoside resistance genes). That agreed with recent clinical work which showed that endogenous patient skin flora, particularly *Staphylococcus* spp., often accounted for surgical‐site infections and carried distinctive resistance gene repertoires [45, 47].

The ft‐H3 sample′s relative shift toward *Bacteroidetes* (and separation from catheter/MSA clusters) aligned with wound microbiome studies reported greater taxonomic and functional heterogeneity across soft‐tissue sites, with *Bacteroidetes* frequently presented in wound‐associated microbiomes [46]. The distinct positioning of the Trypto samples (negative PC2) suggested an alternative ARG profile, potentially reflected with metabolic or stress–response genes rather than canonical ARGs, consistent with comparative metagenomic datasets that reported condition‐specific resistome signatures [48]. Together, these findings reinforce recent evidence that resistome differences are structured both by host anatomical site and microbial community composition. They highlight that ARG clustering in PCA space reflects biologically meaningful processes, including polymicrobial biofilm formation and the mobilome‐driven spread of resistance genes [49, 47].

The *Proteobacteria*/ARG signal in catheter samples showed the need for targeted antibiofilm strategies, like device coatings or localized antimicrobial delivery. For surgical sites dominated by *Staphylococcus*, interventions could focus on perioperative decolonization as suggested by resistome data. Finally, because wound/skin sites showed more heterogeneity, routine metagenomic profiling could help guide personalized wound management [45, 46]. Many recent studies emphasize the importance of shotgun metagenomics (rather than 16S/Sanger alone) to achieve more complete resistome resolution. Applying deeper sequencing and longitudinal sampling would clarify whether the observed PCA clusters are stable over time and whether ARGs are resident or transient. Experimental validation using culture‐based ARG host assignment and plasmid reconstruction would further substantiate the transfer potential suggested by our PCA vectors [48, 49]. Usual cause of infections were the production of toxins by microorganisms [50]. Mostly, wound infections had been due to biofilm development. In normal wounds, planktonic bacteria were successfully treated and less likely cause disease to develop. When microorganisms in the wound developed biofilms and were stabilized by extracellular polymeric substances, proteins maintained and sustained inflammation and delay wound healing. Because of the polymicrobial nature of biofilms in chronic wounds, nonvirulent bacteria became virulent, damaging host tissue and become challenging to treat. Those wounds exhibited resistance to different antibiotics.

The antibiotic resistance of various bacterial species, including *S*. *aureus* and *E*. *coli*, revealed a trend toward widespread multidrug resistance, particularly to beta‐lactams, fluoroquinolones, and aminoglycosides (Table S1). Antibiotic resistance has led to increased multidrug resistance (Figure 4) driven by genetic mechanisms such as efflux pump overexpression, target site modifications, and gene amplification. These mechanisms could complicate treatment and reduce antibiotic efficacy. Alternative strategies such as combination therapies and the use of efflux pump inhibitors could enhance treatment outcomes by overcoming resistance mechanisms and restoring antibiotic effectiveness.

Figure 3(a) Biofilm formation by tube method, wells showing strong, moderate, and weak biofilm formation in different wells by the test tube and (b) the microtiter plate method optical density of biofilms at 620 nm.

**Figure:**
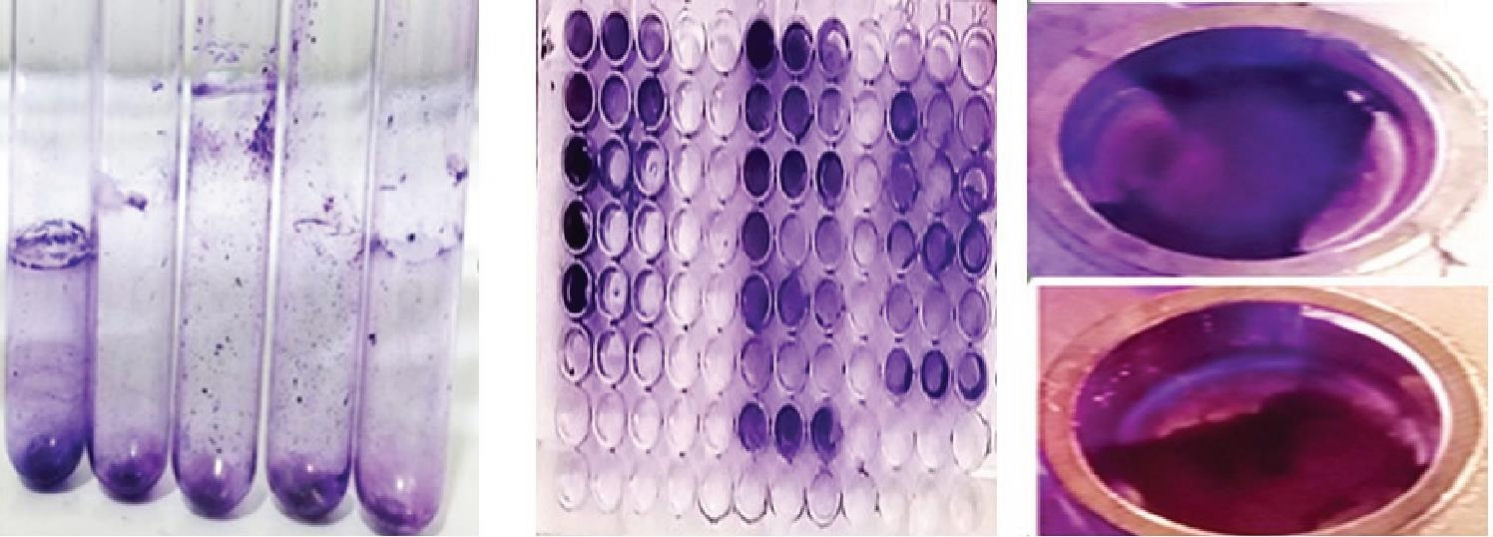
(a)

**Figure:**
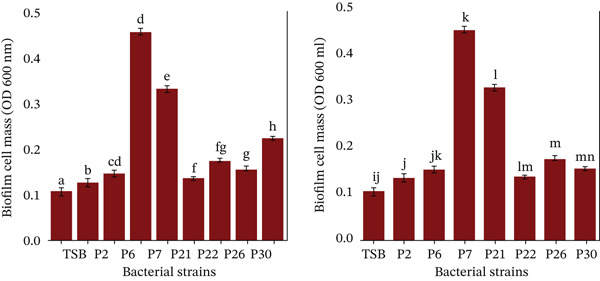
(b)

Figure 7(a, b) The bar chart illustrates the gene expression levels of the *FNBA* gene and clfA gene, using the gyrase gene as a reference.

**Figure:**
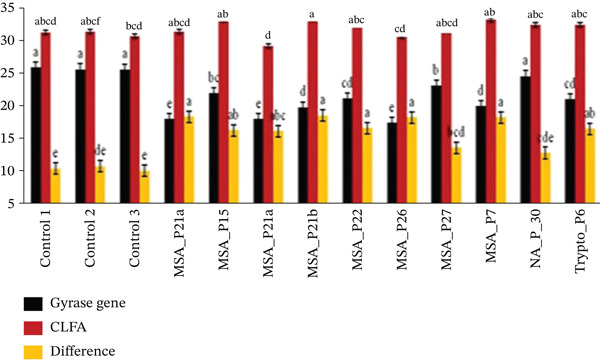
(a)

**Figure:**
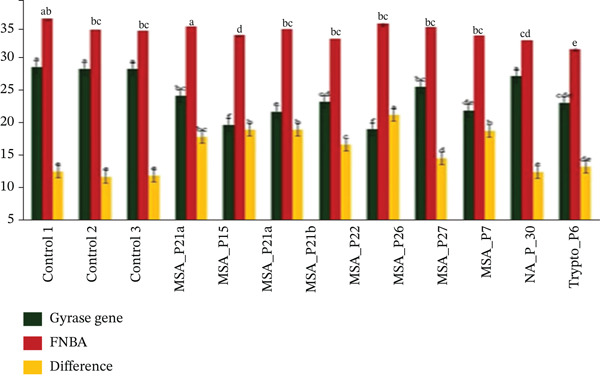
(b)

Figure 9(a) The graphical data illustrate the distribution of phylum abundance across the samples. (b) The bar chart above displays the species‐level abundance in the samples.

**Figure:**
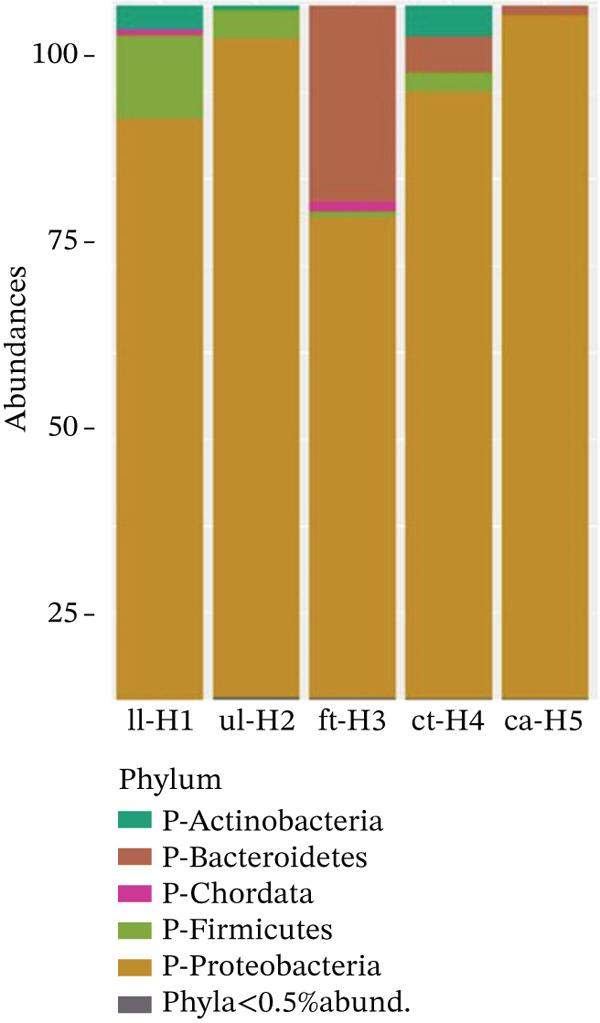
(a)

**Figure:**
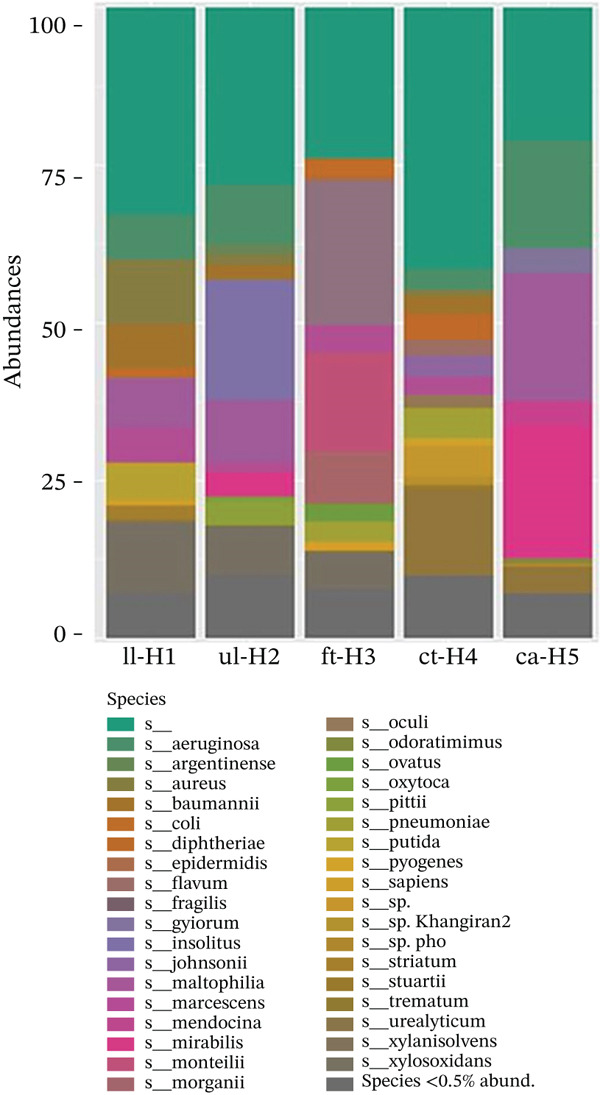
(b)

Figure 10(a) This figure represents a principal coordinates analysis (PCoA) plot based on Jaccard and (b) Bray–Curtis distances among five groups (G1–G5), illustrating the differences in community composition along the first two principal coordinates (PC1 and PC2).

**Figure:**
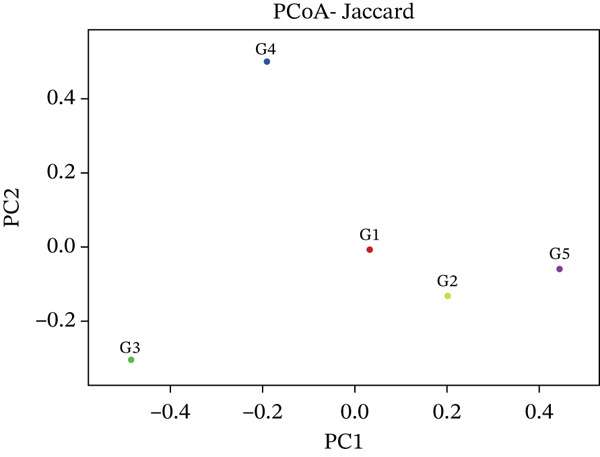
(a)

**Figure:**
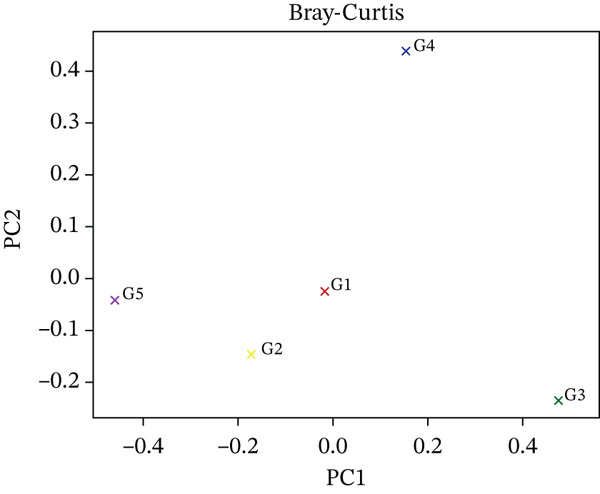
(b)

Our findings were consistent with prior studies that have highlighted *P*. *aeruginosa*, *S*. *aureus, and K*. *pneumoniae* as dominant biofilm‐forming pathogens in nosocomial infections [51, 52]. Notably, our identification of *A*. *xylosoxidans* and *B*. *trematum* in chronic wound infections provided new insights into lesser known but clinically significant pathogens and suggested that current diagnostic protocols might overlook emerging threats. This expanded the known spectrum of nosocomial pathogens and underscored the need to update surveillance frameworks.

Clinically, the results had significant implications for infection management and control. The identification of high levels of ARGs in biofilm‐associated pathogens reinforced the importance of routine resistance profiling in hospitals, particularly in low‐resource settings where empirical treatment was common. Metagenomic analysis could be integrated into diagnostic workflows to rapidly detect both pathogens and resistance genes, thereby improving treatment specificity and reducing the misuse of antibiotics. Furthermore, knowledge of dominant biofilm‐associated species could inform the development of targeted antibiofilm therapies, enhanced catheter and implant coating technologies, and refined disinfection protocols to prevent persistent colonization in surgical environments. Ultimately, such strategies could reduce the incidence and chronicity of SSIs and improve patient outcomes.

Approximately 80% of SSIs involved biofilm‐forming bacteria, and studies from certain African regions highlighted the specific resistance patterns, that is, penicillin and ampicillin resistance. Study conducted on monofilament and braided sutures revealed greater prevalence of *S*. *aureus* a reported multidrug resistant organism [53], penicillin and ampicillin resistance being dominant in the strains. Our study uniquely identified and emphasized the critical roles of specific biofilm‐related genes in *S*. *aureus*, particularly the icaADBC operon, which is essential for polysaccharide intercellular adhesin (PIA) production, and the Agr system, which regulates biofilm maturation.

The microbial communities and structure, especially those of bacteria, had been important for treating the disease due to their role in pathogenesis. The microbial communities were diverse and could play a role in both beneficial and harmful effects. However, the abundance of microbial communities was affected by the microenvironment as well as by ecological factors [54]. In this study, the possible effects of bacteria on different patients were investigated. The samples collected were chosen on the basis of the different organs and their effects on different microenvironments.

Generally, variations at the phylum level might provide a better indication of shifts in community structure in response to changes in the microenvironment along with changes in organs. In total, low‐abundance bacterial communities were the main drivers of general responses at the phylum level [55]. The prominent isolates were representatives of a “microbial signature” of disease. Gram‐negative aerobic bacteria were the dominant bacteria in all the groups. Our results were similar to those of Yallew et al. [52], who reported that *S*. *aureus*, *P*. *aeruginosa*, and *Klebsiella* species were the most common pathogens in various parts of the world. The evaluation of microbiological patterns of hospital acquired infections across Europe and Asia resulted in documenting the same three bacteria as a major source of HAIs [51].

This manuscript highlights several key biofilm‐related genes crucial for the formation and stability of *S*. *aureus* biofilms. Notably, the icaADBC operon is essential for producing PIA, which promotes cell‐to‐cell adhesion. Other significant genes include *clfA* and *clfB*, which facilitate surface adhesion, and *FNBA* and *fnbB*, which enhance adherence to host tissues. Additionally, the Agr system plays a critical role in regulating biofilm maturation and dispersal through quorum sensing, underscoring the complex genetic mechanisms that contribute to *S. aureus* pathogenicity in clinical settings.

Our study had uncovered how biofilms contribute to infection persistence and antimicrobial resistance and highlighted key genetic factors and interspecies interactions that influence pathogenicity. Metagenomic studies also facilitated real‐time ecological insights and guided therapeutic strategies by characterizing biofilm‐associated virulence genes and microbial dynamics in various environments, including clinical settings.

## 5. Limitation of the Study

A limitation of this study included the designing of mono‐strain biofilms, whereas in natural infections complex polymicrobial biofilms are also present. Individual strains exhibit distinct characteristics on their own, but their behavior and pathogenicity can change significantly when combined with other species or factors. This limitation means that the combined effects of multiple pathogens within biofilms should be assessed in the future under standard laboratory conditions.

## 6. Conclusion

Here, we determined the bacterial diversity within biofilms at the molecular level. Culturing techniques were not sufficient to identify the major population within the infection zone, especially anaerobic bacteria. The standard culturing technique examines less than 10% of the bacterial populations from such habitats, so we accompanied our study with the culture‐independent method. As the diagnostic strategies for treating biofilm‐associated infections relies on early detection, utilizing advanced in vitro models to assess biofilm formation and antimicrobial susceptibility would help in identifying treatment options against specific pathogens The current approach provided a comprehensive profile of microbial communities by capturing a wide range of microorganisms, including both aerobic and anaerobic species. This enabled a more complete understanding of infection‐related microbiota by overcoming the limitations of traditional culture methods and leveraging high‐throughput sequencing techniques to detect diverse and often unculturable microbes. The regular assessment of the environmental factors contributing to the biofilm formation was essential, particularly in hospital settings, to aid in the early detection and prevention of infections.

## 7. Future Directions

Future research on biofilm‐associated infections should focus on several key areas to enhance understanding and treatment strategies. One promising avenue is the functional study of identified resistance genes, such as *FNBA*, *clfA*, and *adeFGH*, which were detected in this study and are associated with key biofilm and resistance mechanisms. These gene targets could inform the development of antibiofilm therapies and enhance diagnostic precision. Investigating the genetic and molecular pathways involved in biofilm formation and maintenance will provide insights into potential therapeutic targets. Additionally, exploring the interplay between biofilms and host immune responses can inform strategies to disrupt biofilm persistence and enhance treatment efficacy. Research should also prioritize the development of novel antibiofilm agents and innovative drug delivery systems, such as nanoparticles, to improve the effectiveness of existing therapies. Finally, advancing diagnostic techniques like polymicrobial biofilm to accurately detect biofilm‐associated infections in clinical settings will be crucial for timely and effective management.

## Author Contributions

Haleema Sadia: experimentations and analysis and write‐up; Arshia Amin: project design, hypothesis, result analysis, write‐up, and supervision; Iftikhar Ahmed: project design, supervision; write‐up, and proofreading.

## Funding

No funding was received for this manuscript.

## Conflicts of Interest

The authors declare no conflicts of interest.

## Supporting Information

Additional supporting information can be found online in the Supporting Information section.

## Supporting information


**Supporting Information 1** Table S1: This table presents the detailed antibiotic resistance profiles of clinically isolated bacterial species, including *Staphylococcus, Klebsiella, Proteus, Enterococcus, Escherichia, Pseudomonas,* and *Serratia*, against a broad panel of antibiotics.

**Supporting Information 2** Table S2: This table provides comprehensive metadata of wound infection samples, including sample IDs, source types (swab/tissue), clinical covariates, sequencing approaches (16S rRNA amplicon or shotgun metagenomics), and corresponding NCBI SRA accession numbers. These supplementary datasets support the interpretation of antimicrobial resistance patterns and metagenomic sequencing results discussed in the main text.

## Data Availability

The data that support the findings of this study are openly available in NCBI at https://www.ncbi.nlm.nih.gov/bioproject/?term=PRJNA1064236, Reference Number See Table S2.
