# Supplementary material for: Metagenomic and Phenotypic Insights Into Biofilm‐Forming Pathogens in Patients With Nosocomial Sepsis
Source: Biomed Res Int. 2026 Apr 20;2026:8989667. doi: 10.1155/bmri/8989667 (PMC13096686; doi:10.1155/bmri/8989667)
Supplement: Supplementary file 1 — Supporting Information 1 Table S1: This table presents the detailed antibiotic resistance profiles of clinically isolated bacterial species, including Staphylococcus, Klebsiella, Proteus, Enterococcus, Escherichia, Pseudomonas, and Serratia, against a broad panel of antibiotics. [file BMRI-2026-8989667-s001.docx]

**Supplementary Table S1** Antibiotic Resistance Profiles of Common Pathogenic Bacteria

| **Antibiotics** | ***Staphylococcus* sp.** | ***Klebsiella* sp.** | ***Proteus* sp.** | ***Enterococcus* sp.** | ***Escherichia* sp.** | ***Pseudomonas* sp.** | ***Serratia* sp.** |
| --- | --- | --- | --- | --- | --- | --- | --- |
| Amoxil | R | R |  |  |  |  |  |
| Penicillin | R |  |  |  |  |  |  |
| Augmentin | R | R |  |  |  |  |  |
| Minocycline |  |  |  |  |  |  |  |
| Doxcycline |  |  |  | R |  |  |  |
| Tigecycline |  |  |  |  | R |  |  |
| Erythrocin | R |  |  |  |  |  |  |
| clindamycin |  |  |  |  |  |  |  |
| Gentamycin |  |  |  |  | R | R |  |
| Amikacin |  |  | R | R | R |  |  |
| Methicilin | R |  |  |  |  |  |  |
| ciproxin | R |  |  |  |  |  |  |
| enoxacin | R |  |  |  |  |  |  |
| ofloxacin | R |  |  |  |  |  |  |
| Levofloxcin | R |  |  |  | R | R |  |
| Moxifloxacin | R |  |  |  | R | R |  |
| Cotrimoxazole | R |  |  | R | R | R |  |
| Ceftriaxone |  | R |  |  |  |  |  |
| Tazobactam+pipracin |  |  | R |  |  |  |  |
| Cefoparazone |  |  | R |  |  |  |  |
| Fosfomycin |  |  | R |  |  |  |  |
| Cefipime |  | R | R |  | R | R |  |
| ceftazidime |  |  | R |  |  |  |  |
| Clarithromycin |  |  |  | R |  |  |  |
| Sulphonamides |  |  |  | R |  |  |  |
| Tetracycline |  |  |  | R |  |  |  |
| Ampicilim-sulbactam |  | R |  |  | R | R | R |
| Amoxicillim |  |  |  |  | R |  | R |
| Cephalexin |  |  |  |  | R |  |  |
| Cefpodoxime |  |  |  |  | R |  | R |
| Ciprofloxacin |  |  |  |  | R |  |  |
| Minocycline |  |  |  |  | R |  |  |
| Meropenem |  |  |  |  |  | R |  |
| Cephradine |  |  |  |  |  |  | R |
| Polymyxin b |  |  |  |  |  |  | R |
| Cefoxitin |  |  |  |  |  |  | R |
