# Supplementary material for: Metagenomic and Phenotypic Insights Into Biofilm‐Forming Pathogens in Patients With Nosocomial Sepsis
Source: Biomed Res Int. 2026 Apr 20;2026:8989667. doi: 10.1155/bmri/8989667 (PMC13096686; doi:10.1155/bmri/8989667)
Supplement: Supplementary file 2 — Supporting Information 2 Table S2: This table provides comprehensive metadata of wound infection samples, including sample IDs, source types (swab/tissue), clinical covariates, sequencing approaches (16S rRNA amplicon or shotgun metagenomics), and corresponding NCBI SRA accession numbers. These supplementary datasets support the interpretation of antimicrobial resistance patterns and metagenomic sequencing results discussed in the main text. [file BMRI-2026-8989667-s002.docx]

**^Supplementary Table S2.^** ^Details of wound infection samples collected from post-surgical patients in orthopedic and surgery wards. The table lists sample IDs, sources (swab/tissue), associated clinical covariates, sequencing approach (16S rRNA amplicon or shotgun metagenomics), and corresponding accession numbers.^

| **(Sample ID** | **SOURCE** | **Covariates** | **Sequencing Type** | **Accession** |
| --- | --- | --- | --- | --- |
| H11 | Wound swab | Orthopedic ward, post-surgical | 16S rRNA amplicon | SRR27533909 |
| H12 | Wound swab | Surgery ward, post-surgical | 16S rRNA amplicon | SRR27533908 |
| H13 | Wound swab | Orthopedic ward, post-surgical | 16S rRNA amplicon | SRR27533905 |
| H14 | Wound swab | Surgery ward, post-surgical | 16S rRNA amplicon | SRR27533904 |
| H15 | Wound swab | Orthopedic ward, post-surgical | 16S rRNA amplicon | SRR27533903 |
| H16 | Wound swab | Surgery ward, post-surgical | 16S rRNA amplicon | SRR27533902 |
| H17 | Wound swab | Orthopedic ward, post-surgical | 16S rRNA amplicon | SRR27533901 |
| H18 | Wound swab | Surgery ward, post-surgical | 16S rRNA amplicon | SRR27533900 |
| H19 | Wound swab | Orthopedic ward, post-surgical | 16S rRNA amplicon | SRR27533899 |
| H20 | Wound swab | Surgery ward, post-surgical | 16S rRNA amplicon | SRR27533907 |
| LLH1a | Wound tissue | Orthopedic ward, post-surgical | Shotgun Illumina | SRR27452571 |
| ULH2 | Wound tissue | Orthopedic ward, post-surgical | Shotgun Illumina | SRR27759698 |
| FtH3 | Wound swab | Orthopedic ward, post-surgical | Shotgun Illumina | SRR27454306 |
| Chp4 | Wound swab | Surgery ward, post-surgical | Shotgun Illumina | SRR27485611 |
| CaH5 | Wound swab | Surgery ward, post-surgical | Shotgun Illumina | SRR27448431 |
